# Analysis of prognostic factors for advanced pancreatic neuroendocrine carcinoma: A population-based study

**DOI:** 10.1097/MD.0000000000047285

**Published:** 2026-01-16

**Authors:** Jianyong Zhang, Shimin Zhang, Yang Xu, Xue Wang, Jiang Cheng, Xue Leng

**Affiliations:** a Oncology Department, The First People’s Hospital of Guangyuan, Sichuan Province, China; b Radiation Oncology Department, The First People’s Hospital of Guangyuan, Sichuan Province, China; c Oncology Department, Jian’ge County Hospital of Traditional Chinese Medicine, Sichuan Province, China; d Interventional Therapy Department, The First People’s Hospital of Guangyuan, Sichuan Province, China.

**Keywords:** chemotherapy, neuroendocrine carcinoma, pancreatic tumor, prognosis, surgery

## Abstract

Pancreatic neuroendocrine carcinoma (PNEC) is a rare and aggressive malignancy with poor prognosis. However, large-scale studies on the prognostic factors of PNEC remain limited. We retrospectively extracted clinical data of patients diagnosed with PNEC between 2004 and 2021 from the surveillance, epidemiology, and end results (SEER) database. Overall survival (OS) and cancer-specific survival (CSS) were analyzed using Kaplan–Meier method and compared by log-rank test. Univariate and multivariate Cox proportional-hazards regression models were employed to identify independent prognostic factors. Among 2089 included patients with PNEC, the median age was 61 years (range: 11–90 years). The median OS was 13.0 months, with 1-, 3-, and 5-year OS rates of 51.3%, 29.1%, and 20.1%, respectively. The median CSS was 14.0 months, with corresponding 1-, 3-, and 5-year CSS rates of 53.0%, 31.2%, and 21.9%, respectively. Multivariate analysis identified that advanced age (HR = 1.02, 95% CI: 1.01–1.03, *P < *.001), poor histological differentiation (HR = 1.35, 95% CI: 1.12–1.63, *P* = .002), primary tumor located in the head (HR = 1.28, 95% CI: 1.09–1.51, *P* = .003) or body (HR = 1.42, 95% CI: 1.15–1.75, *P *= .001) of the pancreas, regional lymph node metastasis (HR = 1.32, 95% CI: 1.14–1.53, *P* < .001), absence of primary site surgery (HR = 2.15, 95% CI: 1.82–2.54, *P *< .001), and absence of chemotherapy (HR = 1.87, 95% CI: 1.63–2.15, *P* < .001) were independent prognostic factors associated with worse OS and CSS. Patients with PNEC have poor prognosis with median survival of approximately 13 months. Several clinicopathological factors including age, tumor differentiation, tumor location, lymph node status, and treatment modalities were identified as independent prognostic indicators. These findings provide valuable insights for prognostic stratification and treatment decision-making in patients with PNEC.

## 1. Introduction

Pancreatic neuroendocrine neoplasms (PNENs) are a rare group of tumors originating from pancreatic endocrine cells. Despite sharing anatomical location with pancreatic ductal adenocarcinoma (PDAC), PNENs exhibit a distinct biological spectrum ranging from relatively indolent to highly malignant. This results in fundamentally different clinical prognosis patterns compared to PDAC and dictates divergent therapeutic approaches for the 2 entities. According to the World Health Organization classification, these tumors are primarily divided into 2 main categories: well-differentiated pancreatic neuroendocrine tumor (PNET) and poorly differentiated pancreatic neuroendocrine carcinoma (PNEC).^[1]^ The prognosis of PNENs is highly dependent on pathological grading,^[2]^ with clinical course exhibiting significant heterogeneity across different grades. In contrast, the vast majority of PDAC is inherently poorly differentiated or undifferentiated, exhibiting highly consistent aggressive biological behavior and generally poor prognosis.^[3]^ Fundamental differences also exist in systemic treatment strategies: the standard first-line chemotherapy regimen for high-grade PNENs is etoposide combined with platinum-based agents,^[4]^ whereas PDAC primarily employs FOLFIRINOX or gemcitabine-based chemotherapy regimens.^[5]^ In the broader cohort of PNENs patients, those diagnosed with PNEC account for approximately 10% to 20%.^[6]^

The absence of conventional clinical indicators in the majority of PNEC cases, in conjunction with their high degree of malignancy and aggressive nature, results in approximately 70% of patients presenting with metastases at the time of diagnosis. This category of patients is unable to undergo radical surgical treatment, resulting in a very poor prognosis.^[7]^ The rarity of the disease has resulted in a paucity of large-scale research data concerning the clinicopathological features and prognosis of advanced PNEC. Furthermore, there is considerable controversy surrounding the proposed treatment plan. A plethora of studies have indicated that surgery is not recommended for patients diagnosed with PNEC preoperatively.^[8]^ Conversely, other research indicates that surgery combined with additional therapeutic modalities may improve outcomes for these patients.^[9]^

The Surveillance, Epidemiology, and End Results (SEER) database, which was developed by the National Cancer Institute, consolidates data on the majority of cancer patients in the United States, thus constituting a substantial resource for the study of rare tumors. The present study aims to undertake a retrospective analysis of patient data pertaining to PNEC within the SEER database, with a view to exploring its clinicopathological characteristics, in addition to the adverse factors influencing prognosis in late-stage patients. The objective is to provide a reference value for the treatment and prognosis of this group of people.

## 2. Materials and methods

### 2.1. Data sources

The data was extracted from the SEER database using SEER*Stat 8.4.4 software, and the study focused on patients diagnosed with PNEC between 2004 and 2021. The SEER database is a comprehensive repository of information on a substantial number of cancer patients, anonymised to ensure public accessibility of the data. Consequently, the present study did not require approval from an ethics committee.

### 2.2. Inclusion criteria

The histological type was to be based on the International Classification of Diseases for Oncology, 3rd Edition (ICD-O-3) code, which specifically includes 8002/3, 8012/3, 8013/3, 8041/3, 8246/3. In addition, the disease must be classified as stage IV according to the 7th edition of the American Joint Committee on Cancer TNM staging system. Patients with other tumors or unclear survival times must be excluded.

### 2.3. Study variables and outcomes

The variables extracted in this study encompass multiple aspects, including age, gender, ethnicity, tumor location, histological grade, tumor size, presence of regional lymph node metastasis, site of tumor metastasis, whether the primary site has undergone surgical treatment, whether radiotherapy has been performed, whether chemotherapy has been received, marital status, causes of death related to the tumor, patient survival time, and survival status. The study’s endpoint indicators are the patient’s overall survival (OS) and cancer-specific survival (CSS).

### 2.4. Statistical analysis

In this study, SPSS 27.0 software (Chicago) was utilized for the purpose of conducting a detailed statistical analysis of the data. The count data is presented as absolute values and percentages, i.e., n (%). The Kaplan–Meier method was employed to calculate the survival time, and the survival curve was accordingly delineated. In order to make a comparison between the different survival times, the Log-rank test was utilized. In the preliminary stage of the analysis, the Cox proportional risk regression model was employed to conduct a univariable analysis, and variables with *P* <.2 in the univariable analysis were incorporated into the multivariable analysis. Finally, *P* <.05 was used as the criterion of statistical significance. To address potential multicollinearity, the selection of variables for the multivariable model was guided by clinical relevance to avoid including strongly interdependent factors.

## 3. Results

### 3.1. Baseline characteristics of patients

**Figure 1.**
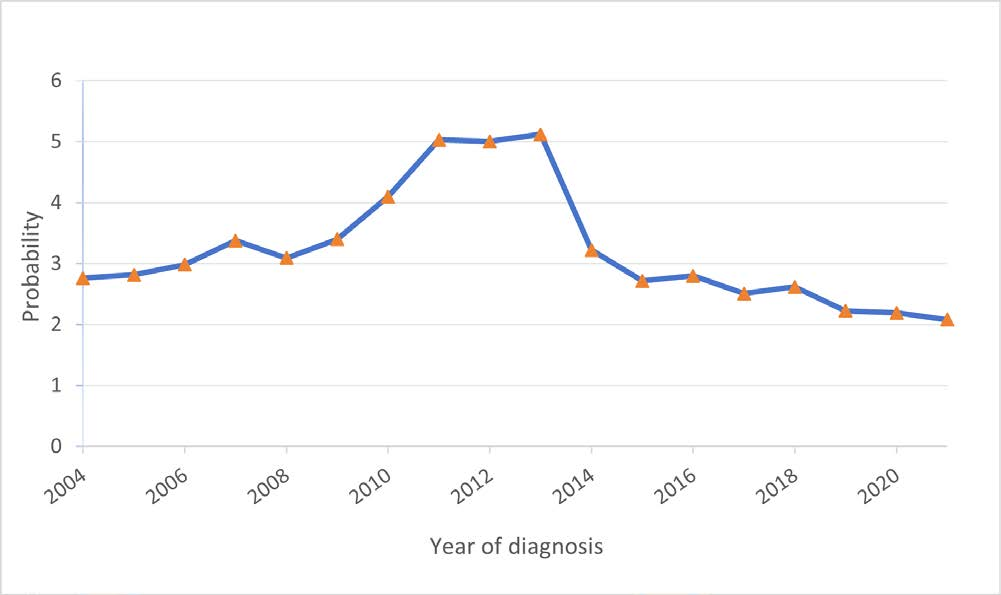
The proportion of pancreatic neuroendocrine carcinoma diagnosed annually in pancreatic malignancies (2004–2021).

**Figure 2.**
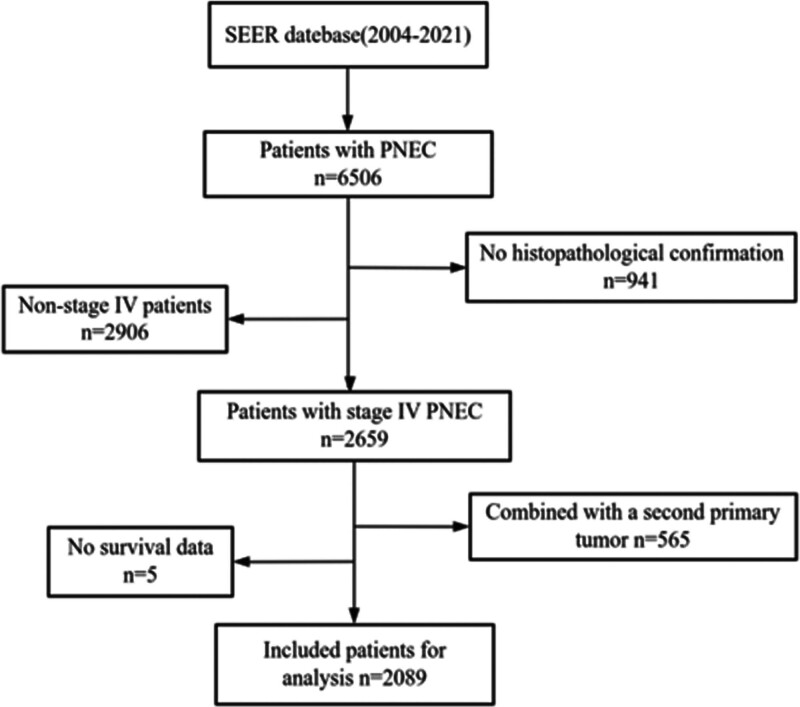
Flowchart for screening PNEC patient data in the SEER database. PNEC = pancreatic neuroendocrine carcinoma, SEER = surveillance, epidemiology, and end results.

Between 2004 and 2021, 6506 patients with PNEC were included in the SEER database, representing 3.17% of all pancreatic malignancies during the same period. The number of patients peaked at 587 cases in 2013 and reached a nadir of 239 cases in 2004. The proportion of PNEC in malignant tumors ranged from 2.08% to 5.12% (Fig. 1). According to the inclusion and exclusion criteria, this study included a total of 2089 patients with a median age of 61 years (range 11–90 years), with the highest incidence rate observed in the 60 to 69 age group (27.7%) (Fig. 2). The basic characteristics of the population are shown in Table 1. This included 1128 male patients (54.0%) and 961 female patients (46.0%), with a male-to-female ratio of approximately 1.17:1. The most common site of tumor occurrence was the pancreatic head, followed by the pancreatic tail. Regarding metastatic sites, the most common site of metastasis was the liver, accounting for 60.3% of all cases. Next were the lungs (8.3%) and bones (7.8%), while brain metastasis was relatively rare (1.4%). In terms of treatment modality, the proportion of patients who underwent surgical treatment was relatively low at 13.5%. However, among patients who received chemotherapy, this proportion was significantly higher at 51.7%. The proportion of patients who received radiotherapy was low at 9.0% (Table 1).

**Table 1:** Basic data of patients with pancreatic neuroendocrine carcinoma.

Variable	N (%)
Overall	2089 (100)
Age	
<50	412 (19.7)
50–59	516 (24.7)
60–69	578 (27.7)
70–79	425 (20.3)
>80	158 (7.6)
Race	
White	1655 (79.2)
Black	261 (12.5)
Other	173 (8.3)
Gender	
Female	961 (46.0)
male	1128 (54.0)
Grade	
G1	232 (11.1)
G2	128 (6.1)
G3	193 (9.2)
G4	76 (3.6)
Unknown	1460 (69.9)
Primary site	
Head	623 (29.8)
Body	221 (10.6)
Tail	562 (26.9)
Unknown	680 (32.6)
Tumor size	
≤4 cm	628 (30.1)
>4 cm	557 (26.7)
Unknown	904 (43.3)
Lymph node metastasis	
No	870 (41.6)
Yes	595 (28.4)
Unknown	624 (29.9)
Bone metastasis	
No	1926 (92.2)
Yes	163 (7.8)
Brain metastasis	
No	2060 (98.6)
Yes	29 (1.4)
Liver metastasis	
No	829 (39.7)
Yes	1260 (60.3)
Lung metastasis	
No	1915 (91.7)
Yes	174 (8.3)
Surgery of the primary	
No	1806 (86.5)
Yes	283 (13.5)
Radiotherapy	
No	1900 (91.0)
Yes	189 (9.0)
Chemotherapy	
No	1008 (48.3)
Yes	1081 (51.7)
Marital status	
Married	1223 (58.5)
Unmarried/widowed/single	781 (37.4)
Unknown	85 (4.1)

### 3.2. The survival of the patient

**Figure 3.**
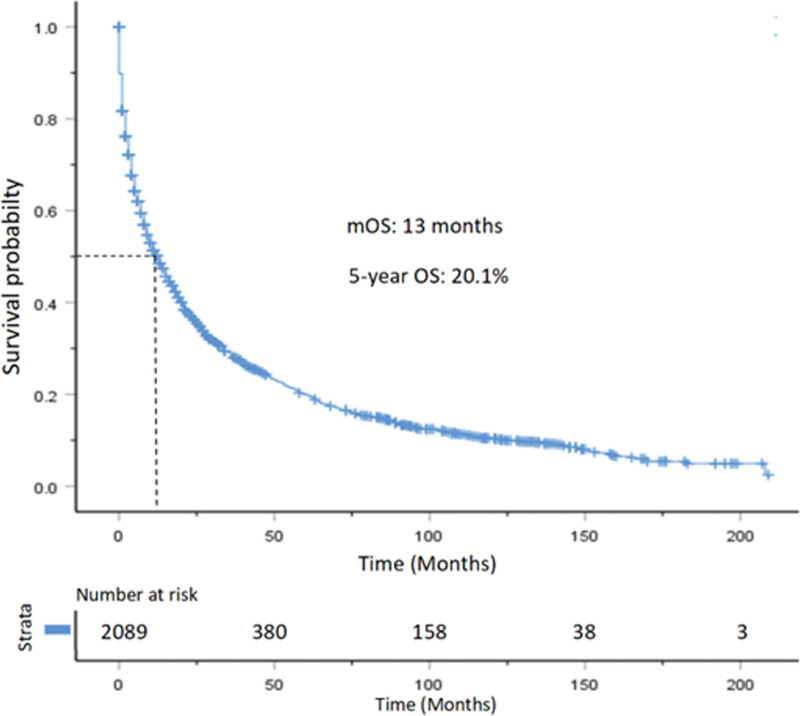
Kaplan–Meier curve for OS in patients with pancreatic neuroendocrine carcinoma. OS = overall survival.

**Figure 4.**
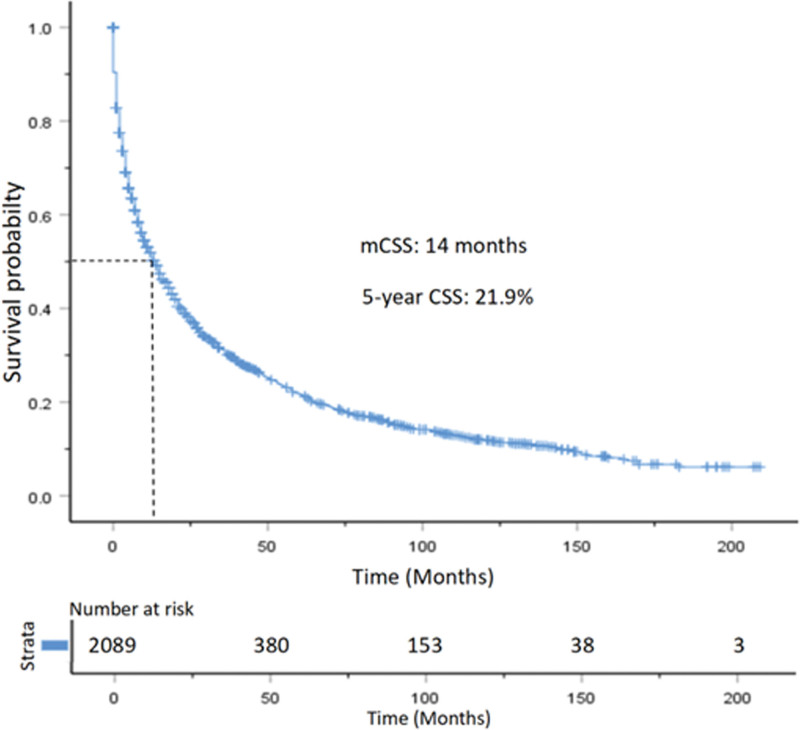
Kaplan–Meier curve for CSS in patients with pancreatic neuroendocrine carcinoma. CSS = cancer-specific survival.

The median OS for the entire study population was 13.0 months, with 1-year, 3-year, and 5-year OS rates of 51.3%, 37.0%, 29.1%, and 20.1%, respectively (Fig. 3). Additionally, the median CSS for the population was 14.0 months, with 1-year, 3-year, and 5-year CSS rates of 53.0%, 39.0%, 31.2%, and 21.9%, respectively (Fig. 4).

**Figure 5.**
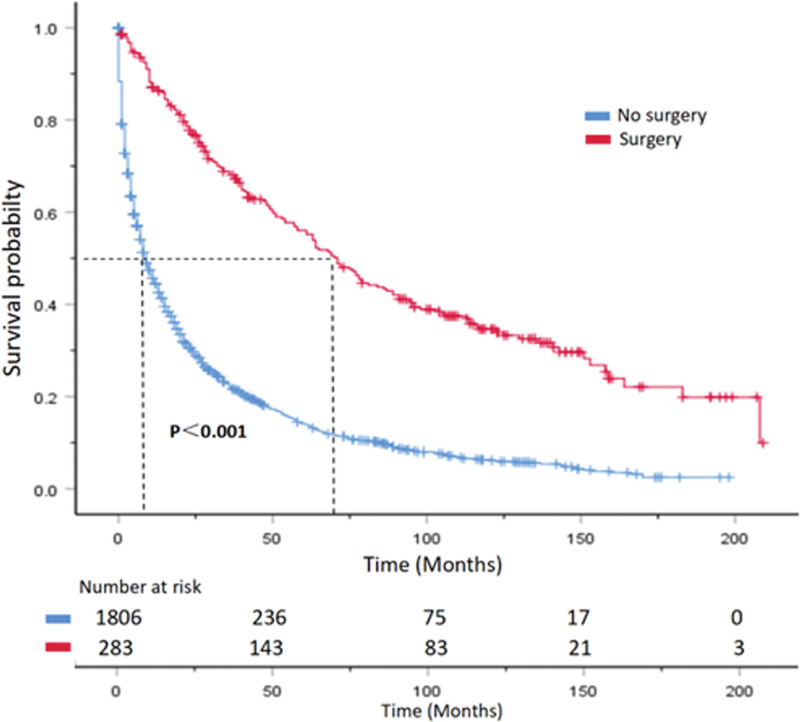
OS curve of PNEC patients with or without surgical treatment. Hazard ratios and 95% confidence intervals were calculated by a stratified Cox proportional-hazards model. OS = overall survival, PNEC = pancreatic neuroendocrine carcinoma.

**Figure 6.**
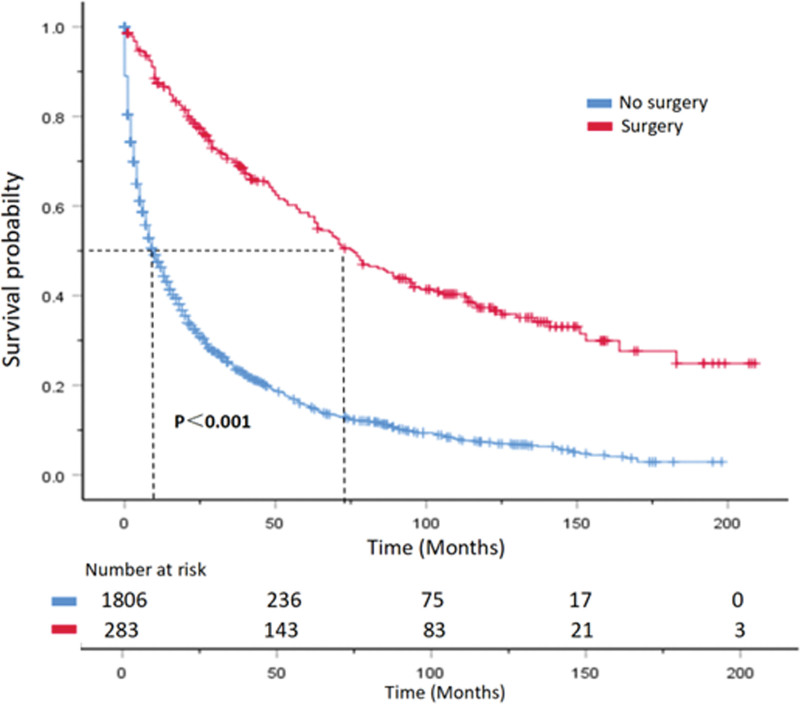
CSS curve of PNEC patients with or without surgical treatment. Hazard ratios and 95% confidence intervals were calculated by a stratified Cox proportional-hazards model. CSS = cancer-specific survival, PNEC = pancreatic neuroendocrine carcinoma.

**Figure 7.**
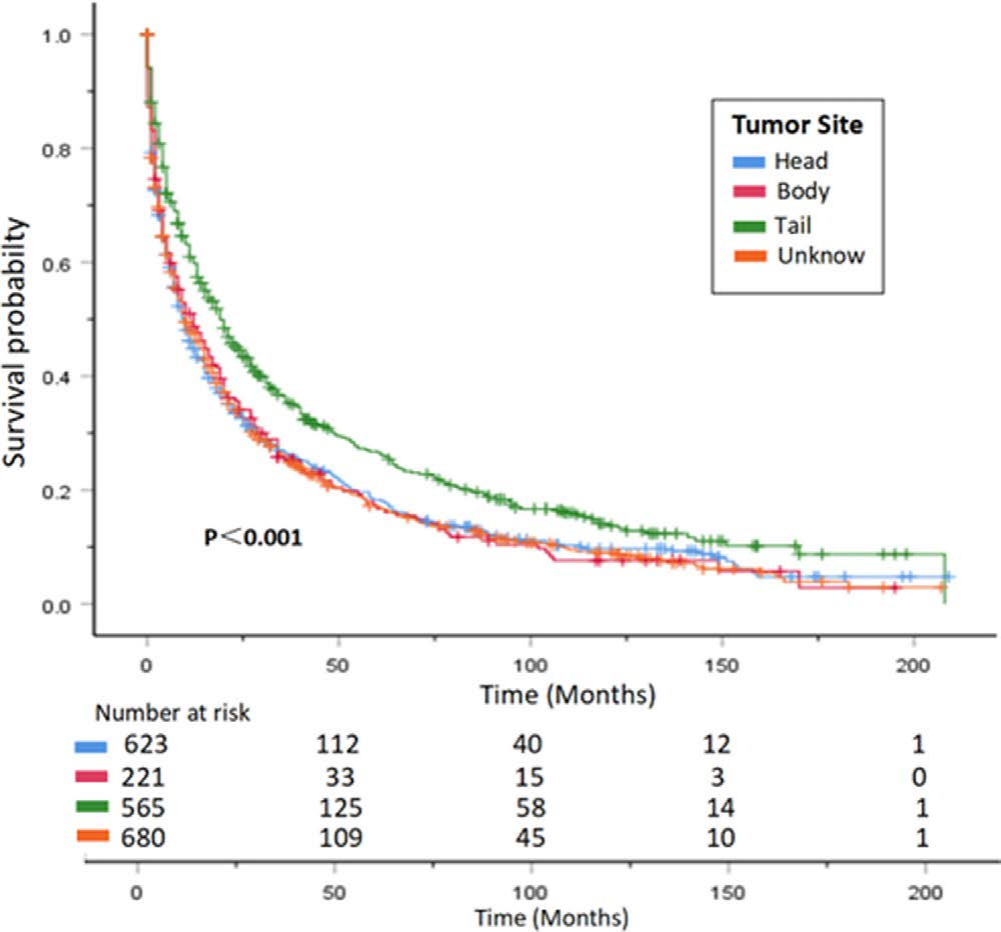
OS curve of PNEC patients with different tumor sites. Hazard ratios and 95% confidence intervals were calculated by a stratified Cox proportional-hazards model. OS = overall survival, PNEC = pancreatic neuroendocrine carcinoma.

**Figure 8.**
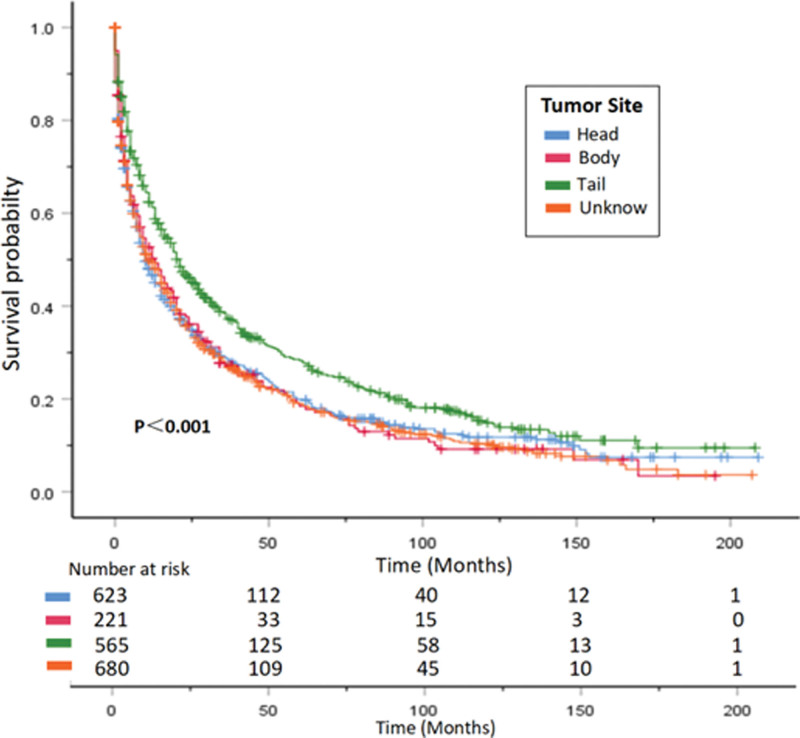
CSS curve of PNEC patients with different tumor sites. Hazard ratios and 95% confidence intervals were calculated by a stratified Cox proportional-hazards model. CSS = cancer-specific survival, PNEC = pancreatic neuroendocrine carcinoma.

The median OS of patients decreases significantly with age, with patients under 50 years old having a median OS of 31 months, while those over 80 years old have a median OS of only 2 months. In histological grading, lower and poorer differentiation are associated with poorer overall survival. Compared with patients with poorly differentiated and undifferentiated tumors, those with well-differentiated and moderately differentiated tumors have a significantly longer median OS (*P < *.001). Regarding tumor location, patients with tumors in the pancreatic tail had significantly better median OS than those with tumors in the pancreatic head or body (*P* < .001). Patients who underwent surgical resection of the primary tumor had a median OS of 71 months, significantly higher than the 9 months for those who did not undergo surgery (*P* < .001). Median CSS also showed a similar trend (Table 2). Survival curves are shown in (Figs. 5–8).

**Table 2:** Median survival time of patients with pancreatic neuroendocrine carcinoma.

Variable	Median OS (mo)	HR (95% CI)	Median CSS (mo)	HR (95% CI)
Age				
<50	31	2.712 (25.685–36.315)	32	3.535 (25.072–38.928)
50–59	16	1.480 (13.100–18.900)	18	1.722 (14.625–21.375)
60–69	10	1.044 (7.954–12.046)	11	1.085 (8.874–13.126)
70–79	6	0.854 (4.326–7.674)	7	0.873 (5.289–8.711)
>80	2	0.621 (0.783–3.217)	2	0.676 (0.676–3.324)
Grade				
G1	49	7.475 (34.349–63.651)	53	8.829 (35.696–70.304)
G2	48	5.998 (36.243–59.757)	48	5.950 (36.338–59.662)
G3	6	0.984 (4.071–7.929)	6	0.984 (4.071–7.929)
G4	5	1.276 (2.498–7.502)	6	1.234 (3.582–8.418)
Unknown	9	0.653 (7.720–102–80)	10	0.752 (8.526–11.474)
Primary site				
Head	10	0.939 (8.159–11.841)	10	1.124 (7.797–12.203)
Body	12	2.286 (7.520–16.480)	13	2.255 (8.579–17.421)
Tail	19	1.838 (15.397–22.603)	21	2.100 (16.885–25.115)
Unknown	10	1.257 (7.536–12.464)	11	1.291 (8.469–13.531)
Lymph node metastasis				
No	15	1.284 (12.482–17.578)	16	1.376 (13.303–18.697)
Yes	17	1.537 (13.988–20.012)	18	1.717 (14.635–21.365)
Unknown	7	0.755 (5.519–8.481)	8	0.808 (6.416–9.584)
Surgery of the primary				
No	9	0.571 (7.880–10.120)	10	0.632 (8.762–11.238)
Yes	71	5.381 (60.453–81.547)	76	6.931 (62.416–89.584)
Chemotherapy				
No	7	1.004 (5.032–8.968)	9	1.475 (6.110–11.890)
Yes	15	0.824 (13.384–16.616)	16	0.886 (14.263–17.737)
Marital status				
Married	15	1.074 (12.895–17.105)	16	1.126 (13.794–18.206)
Unmarried/widowed/single	9	0.807 (7.419–10.581)	10	1.112 (7.821–12.179)
Unknown	17	3.567 (10.009–23.991)	19	3.117 (12.332–15.668)

CI = confidence interval, CSS = cancer-specific survival, HR = hazard ratio, OS = overall survival.

### 3.3. Analysis of factors influencing patient prognosis

The selected variables were included in the Cox proportional-hazards model for univariate analysis. The results indicated that age, pathological grade, primary tumor location, tumor size, lymph node metastasis, bone metastasis, brain metastasis, lung metastasis, primary site surgery, chemotherapy, and marital status were significantly associated with overall survival (*P < *.05). The above factors affecting OS, along with gender and liver metastasis (*P* < .2), were included in the Cox proportional-hazards regression model for multivariate analysis. The results showed that older age (over 50 years), poor histological differentiation, primary tumor located in the head or body of the pancreas, regional lymph node metastasis, no surgical intervention at the primary site, no chemotherapy, brain metastasis, lung metastasis, and unmarried status were independent adverse factors affecting OS (*P* < .05). Detailed results are presented in (Table 3). The same methods were used for univariate and multivariate analyses of CSS (Table 4).

**Table 3:** Univariate and multivariate analysis of OS in patients with pancreatic neuroendocrine carcinoma.

Variable	Univariable analysis		Multivariable analysis	
	HR (95% CI)	*P*-value	HR (95% CI)	*P*-value
Age				
<50	Ref.			
50–59	1.442 (1.244–1.671)	<.001	1.151 (0.990–1.338)	.067
60–69	1.798 (1.557–2.075)	<.001	1.460 (1.262–1.690)	<.001
70–79	2.239 (1.923–2.608)	<.001	1.742 (1.489–2.038)	<.001
>80	3.721 (3.046–4.545)	<.001	2.641 (2.146–3.250)	<.001
Race				
White	Ref.			
Black	1.112 (0.965–1.282)	.141		
Other	0.968 (0.814–1.151)	.71		
Gender				
Female	Ref.			
male	1.075 (0.978–1.181)	.133	1.087 (0.985–1.199)	.097
Grade				
G1	Ref.			
G2	1.230 (0.962–1.574)	.098	1.298 (1.013–1.661)	.039
G3	3.414 (2.756–4.229)	<.001	3.156 (2.534–3.930)	<.001
G4	3.958 (2.990–5.241)	<.001	3.446 (2.585–4.593)	<.001
Unknown	2.564 (2.173–3.024)	<.001	1.865 (1.570–2.216)	<.001
Primary site				
Head	Ref.			
Body	0.991 (0.840–1.171)	.919	0.986 (0.834–1.166)	.871
Tail	0.753 (0.664–0.854)	<.001	0.838 (0.738–0.953)	.007
Unknown	1.014 (0.902–1.141)	.815	1.018 (0.901–1.150)	.774
Tumor size				
≤4cm	Ref.			
>4cm	0.905 (0.798–1.028)	.125	1.013 (0.889–1.155)	.846
Unknown	1.213 (1.085–1.356)	.001	0.951 (0.845–1.072)	.411
Lymph node metastasis				
No	Ref.			
Yes	0.938 (0.837–1.052)	0.274	1.194 (1.059–1.347)	.004
Unknown	1.384 (1.236–1.550)	<.001	1.251 (1.110–1.410)	<.001
Bone metastasis				
No	Ref.			
Yes	1.291 (1.084–1.537)	.004	1.051 (0.874–1.265)	.596
Brain metastasis				
No	Ref.			
Yes	2.281 (1.546–3.366)	<.001	1.719 (1.136–2.600)	.01
Liver metastasis				
No	Ref.			
Yes	1.086 (0.986–1.195)	.095	1.040 (0.942–1.148)	.439
Lung metastasis				
No	Ref.			
Yes	1.735 (1.468–2.050)	<.001	1.318 (1.105–1.573)	.002
Surgery of the primary				
No	Ref.			
Yes	0.326 (0.278–0.383)	<.001	0.385 (0.322–0.461)	<.001
Radiotherapy				
No	Ref.			
Yes	0.961 (0.817–1.131)	.631		
Chemotherapy				
No	Ref.			
Yes	0.900 (0.819–0.989)	.029	0.790 (0.715–0.874)	<.001
Marital status				
Married	Ref.			
Unmarried/widowed/single	1.202 (1.090–1.326)	<.001	1.207 (1.089–1.339)	<.001
Unknown	0.981 (0.766–1.256)	.879	1.061 (0.827–1.362)	.641

CI = confidence interval, HR = hazard ratio, OS = overall survival.

**Table 4:** Univariate and multivariate analysis of CSS in patients with pancreatic neuroendocrine carcinoma.

Variable	Univariable analysis		Multivariable analysis	
	HR (95% CI)	*P*-value	HR (95% CI)	*P*-value
Age				
<50	Ref.		Ref.	
50–59	1.435 (1.233–1.669)	<.001	1.142 (0.979–1.332)	.092
60–69	1.439 (1.238–1.672)	<.001	1.439 (1.238–1.672)	<.001
70–79	2.194 (1.876–2.567)	<.001	1.708 (1.453–2.007)	<.001
>80	3.646 (2.947–4.481)	<.001	2.619 (2.115–3.242)	<.001
Race				
White	Ref.			
Black	1.076 (0.929–1.247)	.329		
Other	0.962 (0.805–1.150)	.672		
Gender				
Female	Ref.		Ref.	
male	1.092 (0.991–1.203)	.075	1.095 (0.989–1.212)	.08
Grade				
G1	Ref.		Ref.	
G2	1.187 (0.922–1.529)	.184	1.245 (0.965–1.605)	.092
G3	3.413 (2.76–4.243)	<.001	3.112 (2.489–3.890)	<.001
G4	3.894 (2.924–5.185)	<.001	3.351 (2.499–4.494)	<.001
Unknown	2.473 (2.090–2.927)	<.001	1.778 (1.491–2.119)	<.001
Primary site				
Head	Ref.		Ref.	
Body	0.996 (0.840–1.183)	.967	0.997 (0.838–1.185)	.969
Tail	0.767 (0.674–0.873)	<.001	0.854 (0.749–0.974)	.019
Unknown	1.023 (0.906–1.155)	.713	1.027 (0.906–1.164)	.674
Tumor size				
≤4cm	Ref.		Ref.	
>4cm	0.943 (0.828–1.075)	.382	1.057 (0.924–1.209)	.42
Unknown	1.235 (1.101–1.386)	.001	0.970 (0.858–1.097)	.629
Lymph node metastasis				
No	Ref.		Ref.	
Yes	0.944 (0.840–1.062)	.338	1.195 (1.056–1.352)	.005
Unknown	1.376 (1.2251.547)	<.001	1.240 (1.097–1.402)	.001
Bone metastasis				
No	Ref.		Ref.	
Yes	1.327 (1.111–1.584)	.002	1.072 (0.888–1.294)	.467
Brain metastasis				
No	Ref.		Ref.	
Yes	2.407 (1.631–3.551)	<.001	1.797 (1.166–2.723)	.006
Liver metastasis				
No	Ref.		Ref.	
Yes	1.079 (0.977–1.191)	.131	1.033 (0.934–1.144)	.528
Lung metastasis				
No	Ref.		Ref.	
Yes	1.751 (1.476–2.078)	<.001	1.323 (1.104–1.586)	.002
Surgery of the primary				
No	Ref.		Ref.	
Yes	0.318 (0.270–0.376)	<.001	0.368 (0.305–0.443)	<.001
Radiotherapy				
No	Ref.			
Yes	0.993 (0.842–1.171)	.935		
Chemotherapy				
No	Ref.		Ref.	
Yes	0.921 (0.836–1.015)	.098	0.803 (0.724–0.891)	<.001
Marital status				
Married	Ref.		Ref.	
Unmarried/widowed/single	1.163 (1.051–1.287)	.004	1.167 (1.049–1.298)	.004
Unknown	0.932 (0.719–1.207)	.592	1.008 (0.777–1.309)	.95

CI = confidence interval, CSS = cancer-specific survival, HR = hazard ratio.

## 4. Discussion

In this study, we conducted a retrospective analysis of the clinical data of PNEC patients in the SEER database, with a view to investigating the clinical characteristics and prognostic factors. This study identified multiple factors significantly associated with prognosis. advanced age, poorly differentiated tumors (low-grade/undifferentiated), tumors located in the pancreatic head or body, and the presence of lymph node, brain, or lung metastases were all associated with poor prognosis. Conversely, surgical resection of the primary tumor, systemic chemotherapy, and spousal support were identified as protective factors. Furthermore, the analysis did not identify race, gender, or radiotherapy to the primary tumor as independent prognostic factors.

PNEC is characterized by a high degree of invasiveness and a high propensity for distant metastasis. With respect to the age of onset, the present study indicates that the majority of patients are in the 60 to 69 age group, accounting for approximately 27.7%. There are significant differences in prognosis among different age groups. Specifically, the median OS for patients aged 50 to 59 is 16 months, for those aged 60 to 69 is 10 months, and for patients over 80, the median OS is a mere 2 months. As demonstrated in the extant literature, patients with early-onset disease who are under the age of 50 have a more favorable prognosis than those with late-onset disease who are 50 and above.^[10]^ While the study by Krug S et al^[11]^ established age over 60 as a key prognostic marker, our study, utilizing a more granular age stratification, reveals that the risk of poorer outcomes increases continuously with advancing age.

In patients with advanced PNEC, the prognosis is generally poor, with a median OS often reported to be less than 1 year.^[2]^ This is consistent with the median OS of 13.0 months observed in the present study. Regarding metastatic patterns, liver is the most common site of metastasis in PNEC, as supported by previous studies reporting rates of approximately 70%,^[12,13]^ followed by lung and bone metastases, while intracranial involvement remains rare. In our cohort, liver metastasis also accounted for the highest proportion (60.3%), aligning with existing literature. While some studies have suggested that liver metastasis is associated with poorer prognosis in PNEC,^[14,15]^ our analysis did not identify it as an independent prognostic factor. Instead, brain and lung metastases emerged as independent adverse predictors for OS in this real-world population-based study using the SEER database.

The standard management for stage IV PNEC primarily involves systemic therapy. Resection of the primary tumor is generally reserved for symptomatic cases – such as those presenting with jaundice, digestive tract obstruction, or bleeding – to alleviate symptoms and improve quality of life.^[16]^ In the absence of such symptoms, prospective evidence supporting primary tumor resection for survival benefit remains limited, and current North American Neuroendocrine Tumor Society guidelines do not recommend routine surgery for these patients.^[17]^ Nevertheless, several studies have reported longer OS in patients with PNEC who underwent surgical resection of the primary tumor compared to those who did not.^[9,15,18]^ Consistent with these findings, our study showed a marked difference in median OS: 71 months in patients who underwent primary tumor resection versus 9 months in those who did not (*P* < .001). Multivariate analysis further identified primary tumor resection as an independent favorable prognostic factor for OS. It should be noted that only a small proportion of patients (13.5%) in our cohort underwent surgery, which may have influenced the outcomes. Therefore, further prospective clinical studies are warranted to validate the effect of primary tumor resection on survival in patients with advanced PNEC.

Radiotherapy is a local treatment widely used in malignancy. While it has been shown to improve survival in limited-stage PNEC,^[19]^ high-quality evidence regarding its role in advanced disease remains scarce. Consistent with National Comprehensive Cancer Network guidelines, which do not recommend radiotherapy as a standalone treatment for metastatic PNEC,^[20]^ our study found no statistically significant difference in overall survival between patients who received primary site radiotherapy and those who did not (*P* = .631). Therefore, primary site radiotherapy should be carefully considered in advanced PNEC, as it may not confer a survival benefit.

Chemotherapy serves as the cornerstone of treatment for stage IV PNEC. Current guidelines recommend first-line regimens analogous to those for small cell lung cancer, primarily etoposide plus platinum-based agents.^[13]^ This combination has demonstrated efficacy in advanced neuroendocrine carcinoma, with objective response rates around 30% and median OS of approximately 1 year.^[21–23]^ Consistent with these findings, our study revealed significantly longer median OS in chemotherapy-treated patients compared to those without chemotherapy (15 vs 7 months; *P* <.05). Multivariate analysis confirmed that omission of chemotherapy was an independent risk factor for poorer OS. However, as the SEER database lacks detailed information on specific regimens, further clinical studies are warranted to optimize chemotherapy protocols for advanced PNEC.

Immunotherapy has emerged as a key investigational approach alongside chemotherapy in neuroendocrine carcinoma. While immune checkpoint inhibitors have shown variable efficacy across neuroendocrine tumors,^[24]^ emerging evidence suggests that patients with poorly differentiated NEC may represent a subgroup with enhanced responsiveness.^[25]^ Unfortunately, the SEER database does not capture immunotherapy-specific data, highlighting the need for prospective clinical datasets to validate these findings. In the realm of targeted therapy, sunitinib has established efficacy in well-differentiated PNET,^[26]^ though its role in PNEC remains undefined. Promisingly, anlotinib has been associated with survival extending to 60 months in advanced PNEC,^[23]^ and a single-center study reported that combining chemotherapy with targeted therapy may improve survival in PNEC patients with liver metastases.^[27]^ These findings provide a foundation for developing novel combination strategies in this aggressive malignancy.

The association between pancreatic tumor location and prognosis is a topic of significant current research interest. In PDAC, pancreatic head cancer typically carries a better prognosis than pancreatic body-tail cancer, primarily because the latter often presents symptoms at an advanced stage, with most cases diagnosed beyond surgical intervention opportunities.^[28]^ However, this anatomical-prognosis association remains unclear in PNENs. Previous studies generally suggest a weak or no significant correlation between location and prognosis.^[29]^ Notably, a recent study presents a contrasting perspective, indicating that survival rates may actually be higher in PNENs patients with tumors originating in the body or tail of the pancreas compared to those with tumors in the head.^[30]^ This study found that tumor location in the pancreatic head or body constitutes an independent factor for poor prognosis. This phenomenon likely stems from dual anatomical and biological influences. Anatomically, tumors in the pancreatic head frequently cause biliary obstruction, compromising the patient’s systemic condition. Moreover, the standard surgical procedure – pancreaticoduodenectomy – is highly complex and carries significant postoperative complication risks, potentially impacting recovery and survival.^[31]^ However, in this PNEC cohort, the negative effects of pancreatic head/body tumors were more pronounced, suggesting potentially distinct biological behaviors. Biologically, the pancreatic head and body/tail regions exhibit developmental differences during embryogenesis, which may result in distinct molecular characteristics in their adult cells.^[32]^ In PDAC, studies have documented differences in gene mutations (e.g., KRAS) and expression subtype distributions between tumors of ventral and dorsal origin.^[33]^ We hypothesize that similar molecular heterogeneity determined by embryonic origin may also exist in PNEC, potentially influencing its aggressiveness and providing direction for future translational research. The presence of regional lymph node metastasis, a frequent precursor to distant metastasis, has been demonstrated to be associated with a poor prognosis, a finding that has been corroborated by preceding studies.^[34]^ The findings of the present study demonstrate that patients exhibiting combined regional lymph node metastasis are subject to a more unfavorable prognosis, with lymph node metastasis proving to be an independent risk factor with regard to overall survival.

The impact of marital status on cancer is also an increasingly studied topic in the research community. A study on gastric cancer^[35]^ found that unmarried patients had a higher risk of death. The study also noted that marital status may be an important factor in predicting long-term survival rates for patients with early-stage gastric cancer. Recent findings from a study on pancreatic cancer^[36]^ also showed an association between marital status and survival rates in patients with pancreatic ductal adenocarcinoma. Married patients were found to have the most favorable prognosis, while widowed patients were observed to have the most unfavorable prognosis. Our study results align with the above studies, indicating that married patients have better OS than divorced or widowed patients. Multivariate analysis showed that marital status is an independent factor influencing OS. This phenomenon may be attributed to the positive psychological and lifestyle support effects provided by a partner, which can aid in active disease treatment.

Beyond the clinical-pathological factors identified in this study, the molecular characteristics of PNEC provide a biological basis for its aggressive behavior. These tumors frequently exhibit inactivation of key tumor suppressor genes such as TP53 and RB1^[37]^ These alterations are considered hallmark features of poorly differentiated neuroendocrine tumors and represent core drivers of their uncontrolled proliferation and genomic instability. Furthermore, KRAS mutations – a driver gene more prevalent in pancreatic ductal adenocarcinoma – are also observed in some PNEC cases, potentially promoting rapid growth and drug resistance.^[38]^ Conversely, MEN1 mutations, common in well-differentiated pancreatic neuroendocrine tumors, are extremely rare in PNEC, highlighting the distinct genetic origins of these 2 tumor entities.^[38]^ Our prognostic model, developed based on age, differentiation grade, and metastatic patterns, would significantly enhance predictive accuracy if integrated with such molecular data. This approach holds promise for identifying novel therapeutic targets for this devastating disease, paving the way toward more personalized treatment strategies.

This study, based on a clinical cohort predominantly composed of the U.S. population (92% White), concluded that race is not an independent prognostic factor. However, caution is warranted when extrapolating these findings to other global populations, as their generalizability may be constrained by 2 factors: First, variations in the genetic background of different populations. The mutation frequencies and molecular phenotypes of key driver genes (e.g., TP53, RB1, KRAS) in PNEC may vary across racial and geographic genetic backgrounds, potentially influencing disease biology, treatment response, and survival outcomes. Second, significant disparities exist in healthcare systems and clinical practice standards. Survival outcomes in this study were influenced by the specific healthcare environment in the United States, including the availability of early diagnostic technologies, access to standard treatment protocols, drug approval policies, and follow-up systems. Clinical manifestations and prognostic characteristics may exhibit marked heterogeneity across healthcare systems with differing resource allocation, screening strategies, or frontline treatment standards. Therefore, we strongly advocate for international multicenter collaborative studies to collect PNEC data from diverse ethnicities and regions under unified standards. This approach will validate and optimize existing prognostic models, ultimately establishing a truly globally applicable clinical prognostic assessment system to advance precision medicine for this disease.

### 4.1. Limitations

This study yielded a series of significant findings through meticulous analysis of the SEER database. Nevertheless, it should be noted that such an analysis is not without its limitations. Firstly, the SEER database primarily includes data from cancer patients in North America, which may lead to the occurrence of selection bias. Secondly, the time span of patient data is considerable, and the completeness of some information is inadequate. For instance, the absence of radiation dose and the specific nomenclature and dose of chemotherapy drugs can compromise the accuracy of the results. It is important to note that all of these factors have the capacity to exert an influence on the final study results.

## 5. Conclusions

In summary, this study indicates that factors such as age, histological differentiation, primary tumor site, regional lymph node metastasis, history of surgical treatment at the primary site, chemotherapy status, presence of brain or lung metastasis, and marital status are all independent factors influencing patient prognosis. When treating patients with advanced PNEC, these factors must be fully considered.

## Acknowledgments

This research was based on data from the Surveillance, Epidemiology, and End Results (SEER) Program of the National Cancer Institute (NCI). The authors are grateful to the NCI and all contributors to the SEER database. The findings and conclusions in this manuscript are those of the authors and do not represent the views of the NCI.

## Author contributions

**Conceptualization:** Jianyong Zhang, Xue Leng.

**Data curation:** Xue Wang, Xue Leng.

**Formal analysis:** Shimin Zhang, Yang Xu.

**Investigation:** Xue Wang, Jiang Cheng.

**Methodology:** Jianyong Zhang, Xue Wang, Xue Leng.

**Project administration:** Shimin Zhang, Yang Xu.

**Resources:** Shimin Zhang, Xue Leng.

**Software:** Yang Xu, Xue Leng.

**Validation:** Yang Xu, Xue Leng.

**Visualization:** Xue Wang.

**Writing – original draft:** Jianyong Zhang, Xue Leng.

**Writing – review & editing:** Jianyong Zhang, Xue Leng.
